# Combinatorial Regression for Analytical Chemistry

**DOI:** 10.1155/ianc/9740085

**Published:** 2025-12-15

**Authors:** Jens E. T. Andersen

**Affiliations:** ^1^ Department of Chemical and Forensic Sciences, School of Pure and Applied Sciences, Botswana International University of Science and Technology, Plot 10071 Boseja Ward Private Bag 016, Palapye, Botswana, biust.ac.bw

**Keywords:** coefficient of correlation, combinatorial regression, least-squares linear regression, statistics, uncertainty of measurement

## Abstract

A current problem in analytical chemistry is to match the uncertainty of several independent sample replicates with the measurement uncertainty associated with first‐order models. According to contemporary guidelines, calibration lines for method validations should be prepared with few standards, frequently without any blanks. These suggestions weaken the validity of the statistics, which are meant to address truth and accuracy rather than precision. As was previously shown when looking at the pooled calibration (PoPC) paradigm for method validation, excellent precision does not imply good accuracy. A novel technique known as combinatorial regression (CR) was created to estimate slopes, intercepts, and standard deviations to reduce disagreements regarding the computation of measurement uncertainty in accordance with the calibration line. The related standard deviations of several replicates were discovered to be too large to be applied to uncertainty. Concurrently, the idea of employing the numerical values of concentration residuals as promising estimates of measurement uncertainty arose from the fact that the standard deviations of the IUPAC equations are too small to be used in the computation of uncertainties. High‐resolution continuous‐source flame atomic absorption spectrometry (HR‐CR FAAS), which has been demonstrated to produce inconsistent findings for the analysis of elements Na, Mg, and Ni, was used to evaluate the CR method on the determination of copper. The CR provided coefficients of variations (CVs) that rose with concentrations and number of replicates, favoring analyzing low concentrations in contrast to the IUPAC equations.

## 1. Introduction

An alternative method denoted as combinatorial regression (CR) is introduced for calculating slopes and intercepts of straight lines as supplement to least‐squares linear regression (LSLR) that delivers the optimal values of the parameters in terms of vertical residuals or y‐residuals [1]. Although the mathematics of the LSLR is relatively straightforward, understanding the corresponding uncertainties is more challenging to grasp [2], as it remains unclear whether the equations address precision or trueness [3]. The Guide to the Expression of Uncertainty in Measurement (GUM) [4] and the Quantification of Uncertainty in Analytical Measurements (QUAM) [5] addressed the calculation of uncertainties according to the LSLR and weighted LSLR that allegedly provide uncertainties of calibration standards that are comparable to the corresponding uncertainties of sample replicates [6]. The uncertainties of GUM and QUAM are based on the equations of IUPAC [7] that may conveniently also be expressed in matrix formulations [1, 2]. Other methods of LSLR include orthogonal regression (OR) that ought to be the preferred model of calibrations, but it may often be disregarded, owing to the complexity of the uncertainty calculations [8]. The LSLR was widely adopted by the scientific community, owing to a combination of simplicity and lack of alternatives. For the calculations of uncertainties, the law‐of‐propagation of uncertainties was offered in appendix E of QUAM, whereas the IUPAC formula was recommended in the main body of the guideline [5]. The IUPAC formula was endorsed as the ISO standard in 2010 [9], thus serving the professional laboratories as well.

Uncertainty calculations are at the crux of analytical chemistry [10] and, despite efforts to assimilate calculations of uncertainty budgets and standard deviations (SDs) of straight lines [11] to the work, most publications apply the SD of a few replicates to demonstrate trueness as recovery of the methods. However, such an approach merely confuses the building of zero‐order models with first‐order models that may lead to poor estimates of uncertainty of measurements. With very low uncertainties in mind, it becomes difficult to understand how it is possible to obtain recoveries that are close to 100% [12]. It may be speculated why uncertainty calculations according to the straight line have not gained the expected traction, but one good reason would be that the IUPAC formulas predict uncertainties of samples that are smaller than the corresponding uncertainties of standards [7]. This fact makes no sense to the observations in the laboratory where uncertainties of samples are always larger than those of standards, owing to the influence of matrix effects [12]. Furthermore, the uncertainties of IUPAC do not speak of the uncertainty that is related to trueness; they merely inform about the quality of the fitting procedure of the LSLR. Finally, the uncertainty of the LSLR approaches zero when the number of standards increases [7], which essentially hampers reproducibility. Hence, another method [6, 13] is needed for estimating slopes, intercepts, and uncertainties of straight lines because analytical chemistry is supposed to focus on trueness and not on precision [6, 14]. The method of CR is one such approach that is considered in the present study, and it aims to explore the qualities of this approach. The CR is applied to a simulation with 100 data points and examples comprising analysis of copper with HR‐CS FAAS [15] and analysis of a synthetic cannabinoid with QQQ‐MS/MS [13, 16]. Both these technologies are expected to provide highly precise and accurate results, but the implementation of the method showed that this assumption is not always fulfilled, and comprehensive method validation is required to reveal the true performance of the methods [13, 15]. Conditions as fit‐for‐purpose with 6–15 replicates to estimate the uncertainty of measurement and 10 replicates for the determination of the LOD [17] do not fullfil the requirements of comprehensive method validations. Much more effort is required, as proposed by the PoPC where 6‐15 replicates merely represent but a single series of contributions to establish the uncertainty of measurement. Preferably, independent series of measurements without rejection of outliers comprising hundreds of replicates in total provide reliable estimates of the uncertainty of measurement that underpins reproducibility rather than repeatability, which is the genuine goal of analytical chemistry [13, 15].

## 2. Materials and Methods

### 2.1. QQQ‐MS/MS Analysis of Synthetic Cannabinoid JWH‐182

This example is used to illustrate what a calibration line may look like in forensic analysis. Details of the method of analysis have been published earlier, and the present results utilize one of many calibration lines that were used to quantify the synthetic cannabinoid of JWH‐182 [13]. The compound was eluted and identified by HPLC gradient elution in 2% acetic acid in water and methanol gradient, with the methanol proportion starting at 70% and reaching 90% after 7 min. The retention time (*t*_*R*_) was determined as *t*_*R*_ = (6.977 ± 0.040) min (±1SD) after 15 replicates and using a coverage factor of 2 (*k* = 2) for the uncertainty [5]. The JWH‐182 was quantified by QQQ‐MS/MS (electrospray injection micro‐mass Quattro micro QQQ mass spectrometer (Waters)) using the quantifier‐fragment ion at *m/z* = 384.2 amu.

### 2.2. HR‐CS‐FAAS Determination of Copper

The results of the analysis of copper were determined at a wavelength of 324.754 nm with HR‐CS‐FAAS (AnalytikJena GmbH contrAA 700) [15, 18]. The standards were prepared by diluting stock solutions with nitric acid (2% w/w). A solution of 2% nitric acid in distilled water was used as blank, and the same solution was used to prepare standard solutions by dilution of the copper stock solution that was prepared from copper acetate (Merck, Cu (CO_2_CH_3_)_2_, CAS 142‐71‐2) to final concentrations of 0.5 mg/L (5 replicates), 1.0 mg/L (3 replicates), 2.0 mg/L (12 replicates), 4.0 mg/L (9 replicates), 5.0 mg/L (3 replicates), 6.0 mg/L (3 replicates), 8 mg/L (3 replicates), 10 mg/L (9 replicates), 20 mg/L (6 replicates), 40 mg/L (3 replicates), 80 mg/L (3 replicates), and 100 mg/L (3 replicates). A xenon short‐arc lamp was used to generate the incident continuous‐source radiation, and the absorbance was measured with an Eschelle‐type spectrometer that was equipped with a CCD detector. An autosampler (AnalytikJena AS‐F autosampler) propelled the solutions to an air‐acetylene (10:1 ratio) flame that was used for the atomization of the measurands.

## 3. Results and Discussion

### 3.1. Simulation of LSLR Calibration Line With SDs

This example is of practical relevance for guidelines that recommend not including the blank values in the construction of regression lines of calibrations [18, 19]. To get an idea about the uncertainty levels of calibration lines to analytical chemistry, a simulation was investigated using the following MSExcel equation:

(1) Y=X∗0.51.3∗10.1+∗RAND +∗10.2+∗RAND ,

with *X* = 1, 2, 3, …, 100. The simulation produced a straight line with slope ± SD, *b*_1_ = 0.5234 ± 0.0027, and intercept ± SD, *b*_0_ = 1.48 ± 0.16, which were calculated by means of the MSExcel spreadsheet and the AnalysisToolPak add‐in option. The parameters of equation (1) were chosen arbitrarily with the restriction; however, the data should exhibit increasing variations not too large as a function of *X*. This scenario images real situations of analytical chemistry but rarely reported on in the literature [13]. The SDs and associated coefficient of variation (CV%) were computed using the simulation, and they are displayed as a function of the quantity of data that was entered, both from high to low values (Figure 1(b)) and from low to high values (Figure 1(a)). For the case of heteroscedasticity, these computations (Figures 1(a) and 1(b)) show how many data points are needed to achieve low CV values when data are progressively added from either low concentrations and upwards (Figure 1(a)) or from high concentrations and downwards (Figure 1(b)) [20]. The latter method (Figure 1(b)) has some implications for contemporary standards for, for example, pesticide analysis or analysis of medicinal chemicals, even though it is never observed in reality [18, 19]. According to these rules, the calibration lines should be prepared using a small number of standards, excluding the response values of blank solutions. These procedures are equivalent to using condensed versions of method validations, which can result in inaccurate uncertainties and poor reproducibility.

Figure 1The coefficients of variation (CV%) as a function of arbitrary concentrations in the MSExcel simulation with 100 data points (equation (1)). The CV values of the heteroscedastic data were calculated by LSLR according to the standard deviations of slopes (a) and intercepts (b) where the number of data were stepwise increased from low concentrations toward high concentrations (broken line, bottom axis) and from high concentrations toward low concentrations (solid line, top axis). The CV values were vastly different between the forward calculations (left‐hand vertical axis) and reverse calculations (right‐hand vertical axis), owing to the inherent heteroscedasticity of the data set that was generated by the simulation.

**Figure:**
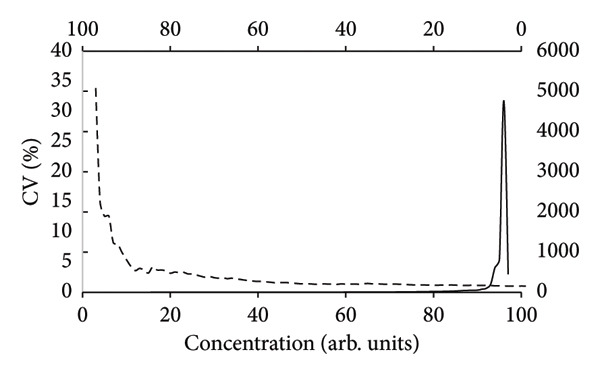
(a)

**Figure:**
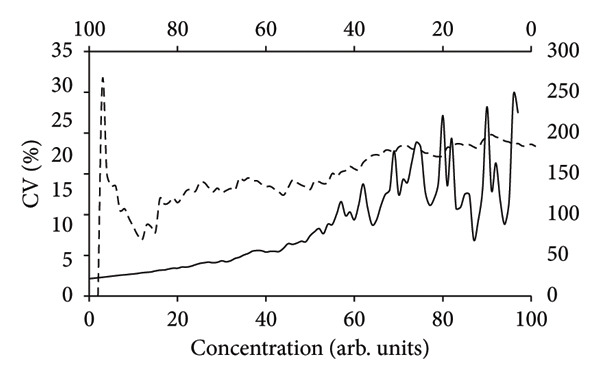
(b)

A calibration line (graph not shown) with heteroscedastic SDs, which are present in many analytical chemistry techniques, was generated by equation (1) [21]. As a result, for both the slopes (Figure 1(a)) and intercepts (Figure 1(b)), a small number of data points with low *X*‐values produced low CV values (Figures 1(a) and 1(b), broken lines), whereas a small number of data points with high *X*‐values produced comparatively large CV values (Figures 1(a) and 1(b), solid lines). While the CV values of slopes and intercepts must be equal after using all 100 data points in the data set, starting with a small number of data points with high concentrations results in excessive CV values of up to roughly 5000 percent for the slopes (Figure 1(a)) and 250 percent for the intercepts (Figure 1(b)). For a calibration line with very small residuals, it should be of no importance whether the calculation of CV values starts from above or below, but in most cases of heteroscedasticity [13], suitable calibration lines may be obtained only by inclusion of a substantial number of data points that, in the present case, amounts to approximately *n* = 20 for the slopes (Figure 1(a)) and more than 70 for the intercepts (Figure 1(b)). Therefore, this simulation shows that before precise estimations of the CV values can be obtained, it may be crucial to include blank values and carry out a thorough method validation in accordance with the PoPC [6]. The simulation thus demonstrates the importance of identifying the degree of heteroscedasticity as a function of concentrations [14] before any conclusions can be made as to the performance of the method. There is a risk of delivering inaccurate uncertainty estimates if the response values between the blank and the standard of lowest concentration are ignored. The simulation also demonstrates that the intercept might not be significant to the calibration line since it is linked to extremely high CV values whenever they are computed after application of a specific amount of data points (Figure 1(b)).

### 3.2. Theory of CR

**Figure 2:**
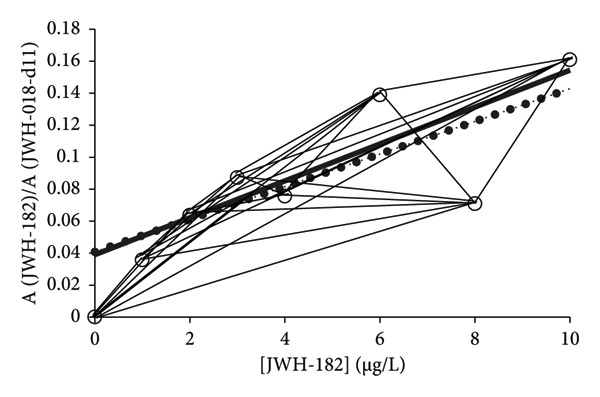
Illustration of the principle of combinatorial regression (CR) with data from an earlier publication treating analysis of synthetic cannabinoid JWH‐182 [13]. The slopes are indicated by thin solid lines connecting two data points only once. With 8 data points, an average of 28 slopes were used to calculate the slope and intercept (not shown) of the calibration line of the CR (solid line). The CR line almost coincided with the calibration line of LSLR (dotted line).

The principle of CR builds on the number of different slopes and intercepts that can be calculated by a combination of any two data points of a data set with more than two data points. That is, for example, two data points provide one slope and one intercept, whereas three data points provide three slopes and intercepts, owing to the number of possible combinations counting each slope and intercept only once. Figure 2 shows a real data set of analysis of a synthetic cannabinoid JWH‐182 [13] with QQQ‐MS/MS where all the possible slopes are indicated by thin solid lines. Consider the simplest case of two data points only that gives two slopes and intercepts of equal magnitude and opposite signs. However, only one is needed, as the degrees of freedom only allow for the data points to be included once in the calculation of the slope and intercept. Hence, the number of slopes and intercepts is reduced to only one for the case of two data points. Similarly, for three data points, the number of independent slopes and intercepts will be three. Therefore, selecting two data points among a group of *n* data points results in the number of combinations (*N*) that can be expressed as a sum, as follows:

(2) N=n!2!·n−2!=12·n·n−1=∑k=1n−1n−k,

where *k* runs through the possible numbers for each value of *n*, which is from 1 to *n* − 1. By using the definition of slope, the sum of slopes (*S*) of data points (*x*_*j*_, *y*_*j*_) and (*x*_*i*+1_, *y*_*i*+1_) may be summed over first single values of *j* followed by summing over *i* running from *j* to *n* − 1, as follows:

(3) S=∑j=1n−1∑i=jn−1yi+1−yjxi+1−xj.

Then, the average value of the slopes (${\overset{¯}{b}}_{1}$) is calculated by means of equations (2) and (3), which yields

(4) b¯1=SN=2n·n−1·∑j=1n−1∑i=jn−1yi+1−yjxi+1−xj.

If the ratio under the sum of (4) were multiplied by (*x*_*i*+1_ − *x*_*j*_) in the numerator and denominator, the expression would correspond to the formula for the slope of the LSLR method [1, 12], which is calculated as the ratio between covariance (COV (*X*, *Y*)) and the variance of concentrations (VAR (*X*)). According to the experiments, the slope of (4) tends to be larger than the corresponding slope of the LSLR.

Accordingly, the average value of the intercepts (${\overset{¯}{b}}_{0}$) is calculated as follows:

(5) b¯0=2n·n−1·∑j=1n−1∑i=jn−1yj·xi+1−yi+1·xjxi+1−xj.

The SDs that correspond to the average values of equations (4) and (5) are then given by the expressions of equations (6a) and (6b), respectively,

(6a) sb¯1=2n·n−1−3·∑j=1n−1∑i=jn−1yi+1−yjxi+1−xj−b¯12, n > 3,

(6b) sb¯0=2n·n−1−3·∑j=1n−1∑i=jn−1yj·xi+1−yi+1·xjxi+1−xj−b¯02, n > 3.

The calculations may also be performed with repetitions that make the difference *x*_*i*+1_ − *x*_*j*_ equal to zero, which provides infinite slopes and intercepts that are simply removed from the data set.

With heteroscedastic data, as in the present case, some of the slopes and intercepts take very large positive and negative values, as illustrated in Table 1 and Figure 2. Proper calibrations are accomplished by using the PoPC, and it is used to demonstrate the degree of data spread, especially at high concentrations, which gives rise to wide ranges and large SDs (Table 1). Therefore, equations (6a) and (6b) cannot be used as measures of uncertainties according to precision, just like the IUPAC formulas of confidence intervals [1, 2, 7] cannot be used as uncertainties either because none of the formulas provides uncertainties that match those of multiple independent replicates of samples [6].

**Table 1:** Comparison of the values of parameters of LSLR and CR with *n* = 100 in the simulation.

Parameter	LSLR (*n* (LSLR) = 100)	CR (*n* (CR) = 4950)
Slope	0.5234	0.52
SD (slope)	0.0027	0.21
CV% (slope)	0.52	39
Range	—	−2.7 to 3.8

Intercept	1.48	2
SD (intercept)	0.16	15
CV% (intercept)	11	910
Range	—	−290 to 310

*Note:* With linear regression, *n* is also equal to 100 but for the CR, the number of possible values is 4950. Within limits of uncertainty, the slopes and intercepts are equal for LSLR and CR.

It is currently unclear whether CR can effectively replace established multivariate methods such as multiple‐linear regression (MLR), principal component regression (PCR), or partial least‐squares regression (PLS) [22], as this determination needs more research. Meanwhile, other techniques like the method of simple interval calculations (SICs) [23] provide a simplified calibration process by using linear regression based on error estimates. The primary goals of these multivariate approaches are to classify data, compensate for matrix effects, and improve measurement accuracy, which is frequently achieved by examining variance–covariance matrices [24].

### 3.3. HR‐CS‐FAAS Analysis of Copper

Earlier, it was realized that the analysis of elements Na, Mg, and Ni was associated with issues of repeatability when it was performed with single calibration lines for the method validation [15]. Only by applying the PoPC to the method validation was it possible to obtain reasonable correspondence between uncertainties that were predicted by the calibration lines and the uncertainties obtained by multiple replicates of the samples [6, 13]. Copper was analyzed with the same technology, and the same type of issue with repeatability was found for the analysis of this element (Figure 3). A total of 69 measurements were made including three or more repetitions (see above), which provided 2100 slopes and intercepts (Table 2) for the calculation of corresponding average values (Equations (4) and (5)). The bulk of the measurements (*n* = 60) were performed at concentrations of 0–20 mg/L, leaving only 9 data points of concentrations above 20 mg/L. The calibration line of LSLR was influenced by the data of high concentrations more than those of lower concentrations, which led to a shallow slope in comparison with that of the CR (Figure 3(a)). According to the PoPC [6], the ULA was determined as 29 mg/L, which corresponds well to the predictions of CR (Figures 3(a), 3(b), 3(c), and 3(d)).

Figure 3Calibration lines of the determination of copper with HR‐CS FAAS and application of LSLR (broken lines) and CR (solid lines) to prepare the corresponding calibration lines. A clear curvature was identified for the data in (a), which shows that the linear range should be found at lower concentrations that was immediately identified by the CR; that is, it is most likely below 20 mg/L. Some curvature remained by using the data of concentrations up to 40 mg/L (b), but no or little curvature was found for the data of concentrations up to 20 mg/L (c). No apparent curvature was found for the data of concentrations up to 10 mg/L (d) where the calibration lines of LSLR and CR coincided. This observation suggests that the gradual approach of the two calibration lines may be used to identify the calibration interval of concentrations that is most suitable for the analysis at the lowest possible uncertainty of measurement.

**Figure:**
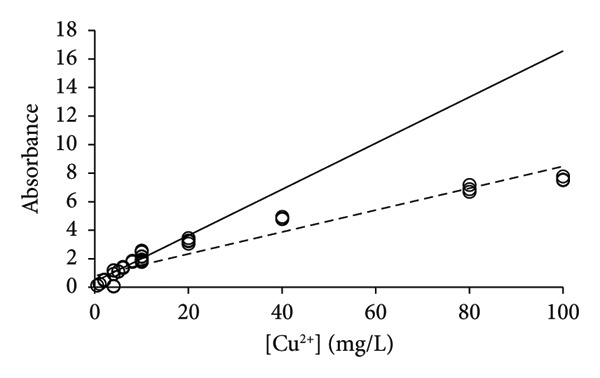
(a)

**Figure:**
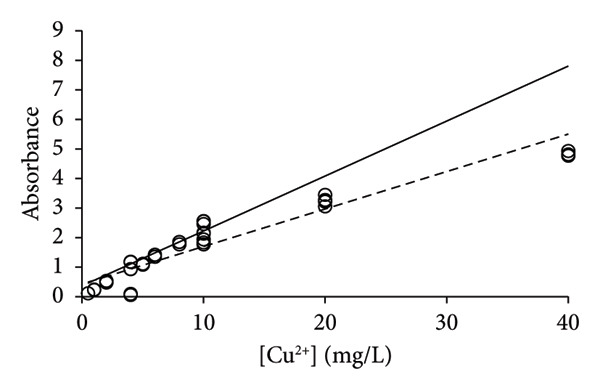
(b)

**Figure:**
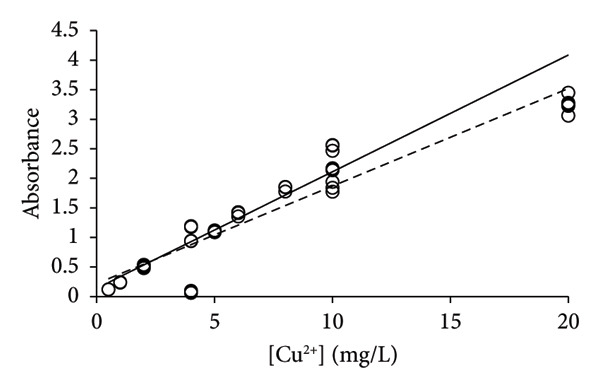
(c)

**Figure:**
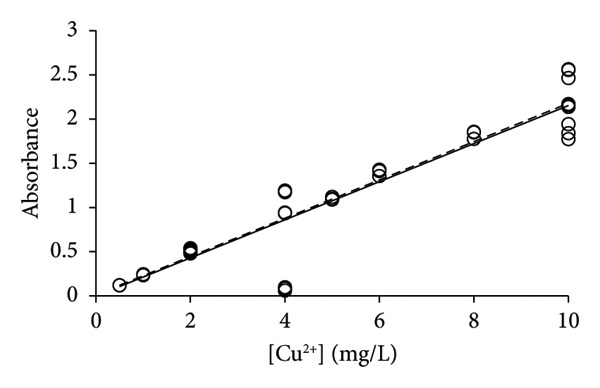
(d)

**Table 2:** Comparison of slopes that were determined by LSLR and the CR for the results of Figure 3.

Max. [Cu^2+^] (mg/L)	*n* (LSLR)	LSLR	CV% LSLR	CR	*n* (CR)	SD CR	CV% CR	SEM CR
2	27	0.251	1.2	0.247	216	0.012	4.9	0.00082
10	54	0.217	4.4	0.20	1035	0.10	52	0.0032
20	60	0.165	3.9	0.20	1428	0.14	69	0.0036
40	63	0.127	4.2	0.19	1449	0.13	71	0.0035
80	66	0.089	4.4	0.17	1860	0.13	76	0.0030
100	69	0.077	3.9	0.17	2100	0.12	69	0.0025

*Note:* This example illustrates the convergence of the values of the slopes as the number of replicates *n* (LSLR) and *n* (CR) decreases. The magnitudes of the SDs of the mean (SEM) of the CR are narrow, owing to the large number of slopes (*n* (CR)) that are involved in the calculations.

**Figure 4:**
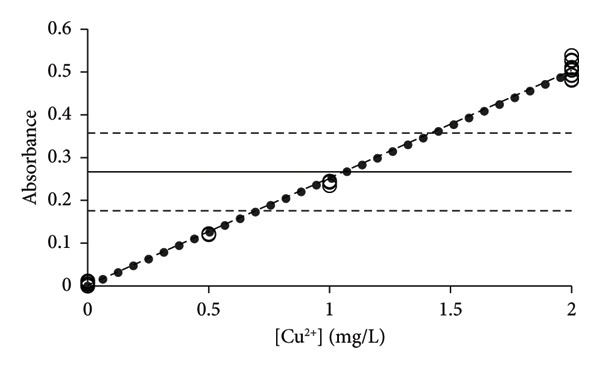
The calibration line (solid line) and confidence intervals (broken lines) for the data are presented in Table 2 and Figure 3. Compared to the calibration lines of Figure 3, this calibration line of CR has only six out of 27 data points inside the limits of the confidence interval of a zero‐order model, which means that the first‐order model prevails.

By excluding data with [Cu^2+^] > 40 mg/L, the new calibration line of CR approached that of the LSLR (Figure 3(b)), and the correspondence between the LSLR improved even further by excluding data of [Cu^2+^] > 20 mg/L (Figure 3(c)) and [Cu^2+^] > 10 mg/L where the correspondence for the latter was excellent (Table 2, Figure 3(d)). These properties of the CR show that already in Figure 3(a), the line indicated that the upper limit of analysis (ULA) [13] should be limited to 20 mg/L or less. Thus, the CR has a built‐in weighting scheme that highlights the most favorable calibration range in terms of the density of data rather than the leverage of the high‐concentration data that characterizes the method of LSLR [21]. The SDs, CV values, and SEMs [25] of the slopes are almost identical for the various ranges of concentrations (Table 2) except for the concentrations below 2 mg/L where the calibration line exhibited marked improvements, but excellent performance for concentrations up to 5 mg/L has also been reported [26]. As it was established earlier for the elements Na, Mg, and Ni, the quality of the results was less than expected for routine types of analysis [6, 15], whereas, for the present case of copper, the 27 data points that were recorded for [Cu^2+^] ≤ 2 mg/L provided excellent calibration lines (Figure 4), with the CV value of only 4.9% to the slope (Table 2). However, this may be a case of sheer coincidence, as the data of higher concentrations exhibited much higher CV values. On the other hand, improved performance at low concentrations might be a special feature of this type of technology. According to the present data, it cannot be ruled out that the preferred calibration interval would be 0 ≤ [Cu^2+^] ≤ 2 mg/L for the determination of copper with the HR‐CS FAAS technology.

To further confirm the validity of the first‐order model, it is necessary to perform a simple test to distinguish between the zero‐order and first‐order models [6, 15] as demonstrated in Figure 4 where the lower and upper limits of the confidence intervals, according to the average response values, are shown to contain much less than the expected 95% of the data. Hence, the first‐order model is most likely the correct model to describe the data. IUPAC [7]and Eurachem [17] recommend a blank, 5‐6 levels for the standards and a few repetitions, which in the present context does not meet the criteria for a full method validation. Frequently, guidelines recommend 5 data points and a few replicates to construct the calibration line [18, 19], which, together with potentially shallow slopes, poses the risk of mistaking the zero‐order model for a first‐order model.

### 3.4. Uncertainty of Measurement

Uncertainty of measurement, as determined by the uncertainty budget [5, 27], the IUPAC method [7], or by the QUAM [5, 6], is the key ingredient of decision‐making in analytical chemistry [4, 5]. However, it has proved difficult to obtain the uncertainty, which is predicted by the calibration lines, to match those of the sample uncertainties of a limited number of replicates [21]. It is a challenging task to obtain the correspondence between predicted uncertainties and observed uncertainties, which are also denoted as bottom‐up and top‐down uncertainties [28], because uncertainties of the first‐order model are to be compared with corresponding uncertainties of a zero‐order model [6]. Even for the first‐order model, which is used to calculate uncertainties of both standards and samples, there are issues with meeting such correspondences because the IUPAC formulas deliver uncertainties of samples that are inherently smaller than the uncertainties of standards of the same concentrations [7]. This is rarely observed in practice, owing to the influence of matrix effects on the sample uncertainties [12]. Furthermore, the uncertainties of the IUPAC equations approach zero for the samples but not for the standards as a function of increasing numbers of replicates, which is also not observed in practice [13, 29]. Therefore, if the scientific method [30] is supposed to prevail, the SDs of the IUPAC formulas [7, 9] are not suited to represent the uncertainty of measurement. Since the development of the Horwitz formula and the PoPC [6, 31], the notion of elevated uncertainties and the influence on calibrations of analytical chemistry have started to appear in the literature [14]. The SDs of the CR are extremely large compared to those of the IUPAC [7] and QUAM formulas [5], but, owing to the large number of slopes and intercepts involved in the calculations (Table 2), the SEM is small, thus indicating well‐determined average values of good precision [32]. Since the focus is trueness and not precision, an alternative measure of uncertainty is required. The trueness represents the consensus value of, e.g., interlaboratory comparisons, whereas precision merely speaks of a single series of experiments [6, 33]. The uncertainties calculated by means of the PoPC showed good correspondence between predicted and observed values [13]. However, to mitigate the issue of vanishing SDs when the number of replicates increases, it may be proposed to use a method of calculating uncertainties that is based on *x*‐residuals rather than *y*‐residuals. Thus, it is suggested to use two times the numerical values of the residuals of concentrations, |*r*_*x*_| = |*x*_*i*_ − (*y*_*i*_ − *b*_0_)/*b*_1_|, and fit a straight line with slope $b_{1}^{\prime }$ and intercept $b_{0}^{\prime }$ to the thus‐determined |*r*_*x*_| values, according to the following equation:

(7) r∧x=b1′·xi+b0′.

**Figure 5:**
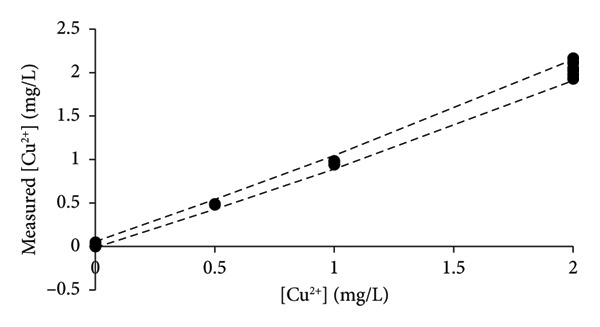
Depiction of measured concentrations (*n* = 27) versus prepared concentrations according to the data of Figure 3 with concentrations from 2 mg/L and below, thus ignoring results of higher concentrations. It is proposed that two times the numerical values of *x*‐residuals |2(*x*_*i*_ − (*y*_*i*_ − *b*_0_))/*b*_1_| should be used as measures of uncertainties in this specific interval of concentrations. Hence, the measured concentrations of unknowns below 2 mg/L ([Cu^2+^] < 2 mg/L) are expected to fall within the limits of the lines that are defined by the magnitude of double residuals (*k* = 2).

By adding $U=\pm 2\cdot \left|{\overset{\wedge }{r}}_{x}\right|$ to the calculated concentrations ($\overset{\wedge }{x_{i}}$), an overview can be presented with the concentrations and corresponding uncertainties depicted as a function of the concentration of standards (*x*_*i*_), which cover most data of the pooled calibrations (Figure 5) [14]. Accordingly, the method validation of the PoPC provides elevated uncertainties, in comparison with current practices and procedures of international guidelines [4, 5]. Developments within the practices of recent guidelines [18, 19] suggest revival of the Horwitz formula [34] that offers elevated CV values that exceed what is normally reported in the scientific literature [27, 35]. It was dismissed for many years, owing to the requirements of uncertainties to be equal to perhaps 10% or less [36] that creates confusion between the concepts of precision and trueness. However, the guidelines now seem to accept CV values of up to 50% for concentrations of 1 mg/kg [18], which is good news because this underpins what is observed in real measurements of quantification of, for example, pesticides [19].

The distribution of slopes for the results of Table 2 and Figure 3 is shown in Figure 6(a) where Scott’s formula [37] was used to calculate the bin widths. Although the data are distributed according to a bell‐shaped curve, it is difficult to tell whether it represents a normal distribution or any other type of distribution that is described by a continuous mathematical function (Figure 6(a)). Many different types of distributions, such as the negative binomial distribution and the normal distribution (Figure 6(a)) [38], can be postulated, each with their own advantages and drawbacks but it may be impossible to decide upon which one of the distributions would be the more favorable one, given the only 69 data points that was used for the present analysis (Figure 3).

Figure 6Distributions of 2076 slopes of the experiments of Figure 3(a) (above) according to (a) the Gaussian distribution with the bin width calculated by Scott’s formula [33] and (b) the canonical distribution with a bin width of one standard deviation. It is proposed that the canonical distribution (b) may be used for comparison of results between laboratories because it does not depend on an underlying mathematical formula.

**Figure:**
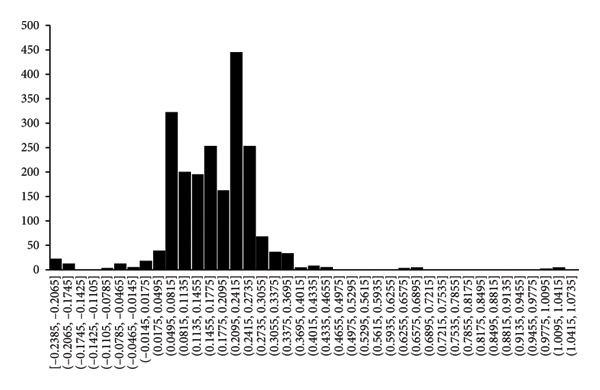
(a)

**Figure:**
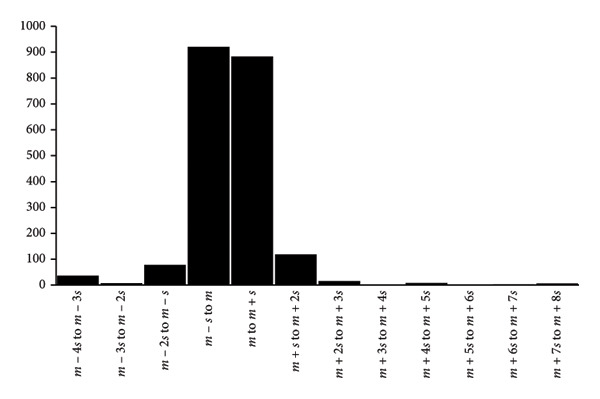
(b)

Instead, it may be proposed to more conveniently use a distribution that is independent of the choice of bin width and thus it does not rely on which formula that best describes the distribution in terms of mathematical formulas and precision. The proposition involves distribution of data with bin widths of ± 1 SD, ± 2 SD, etc., according to the average value, which presents the distribution of Figure 6(b). This distribution (Figure 6(b)) may be denoted as the “canonical distribution” that relates to discrete data only, and it has otherwise no mathematical foundation associated with it. Such a distribution has the benefit of allowing findings from various laboratories to be compared without making any assumptions about the curve’s anticipated shape. To make it easier to compare the findings of different laboratories, the usage of canonical distribution would also encourage users to include more independent data in their statistical analysis.

## 4. Conclusion

For use in analytical chemistry, a novel CR technique for creating calibration lines was created. To compute the relevant SDs and SEMs, the CR offers many slope and intercept values (*N = *1/2^*.*^ *n* (*n* − 1)). The CR has a high degree of precision due to the large number of slopes and intercepts. Additionally, the model weights the magnitudes of slopes and intercepts based on data density rather than the magnitude of data associated with the traditional LSLR technique. It is commonly recognized that neither the LSLR nor the CR uncertainties provide uncertainties that are like sample replicate uncertainties. Consequently, it was suggested that the uncertainty be included as double (*k* = 2) [5] the concentration *x*‐residuals’ numerical values, or $U=\pm 2\cdot \left|{\overset{\wedge }{r}}_{x}\right|$, which showed to cover most of the data points of calibrations. At high concentrations, the residuals from the HR‐CS‐FAAS analysis of copper were very considerable, while at low concentrations 0 ≤ [*C* *u* ^2+^] ≤ 2 mg/L, they were found to be rather small. It is unclear if this was a coincidence or just a feature of the HR‐CS‐FAAS that was used to analyze copper. It is also debatable whether the analysis needs to contain all measurable data for all concentrations or if additional data should be generated to adequately validate the approach.

To confirm the theory of CR, findings from two standard methods of analytical chemistry, HR‐CS‐FAAS and QQQ‐MS/MS, along with a simulation were utilized. While additional data could have been produced, a selective dataset was sufficient to illustrate the core principles of CR for this study.

The current results suggest modifications to the validation protocols for quantification methods in analytical chemistry. The key proposals comprise the following: (1) Never reject outliers, as this practice compromises uncertainty estimates related to trueness and reproducibility. (2) Utilize the CR method to quickly determine the linear range of calibrations. (3) Use the canonical distribution to identify potential double peaks and compare distribution profiles across laboratories, provided that large data sets are analyzed.

## Conflicts of Interest

The author declares no conflicts of interest.

## Funding

This work was supported by the Botswana International University of Science and Technology (BIUST). The research did not receive any specific grant from funding agencies in the public, commercial, or non‐profit organizations.

## Data Availability

The data that support the findings of this study are available from the corresponding author upon reasonable request.
